# Association of preterm outcome with maternal systemic lupus erythematosus: a retrospective cohort study

**DOI:** 10.1186/s13052-023-01436-5

**Published:** 2023-04-01

**Authors:** Xiafang Chen, Wen Di, Lili Ye, Yabin Hu, Meng Jiang, Jiayue Wu, Jun Bu, Jianhua Sun, Fei Bei

**Affiliations:** 1 Department of Neonatology, Shanghai Children’s Medical Center, School of Medicine, Shanghai Jiao Tong University, 1678 DongFang Road, 200127 Shanghai, China; 2 Department of Obstetrics and Gynecology, Ren Ji Hospital, School of Medicine, Shanghai Jiao Tong University, Shanghai, China; 3 Shanghai Key Laboratory of Gynecologic Oncology, Shanghai, China; 4 State Key Laboratory of Oncogenes and Related Genes, Ren Ji Hospital, School of Medicine, Shanghai Cancer Institute, Shanghai Jiao Tong University, Shanghai, China; 5 Department of Rheumatology, Shanghai Children’s Medical Center, School of Medicine, Shanghai Jiao Tong University, Shanghai, China; 6 Department of Clinical Epidemiology and Biostatistics, Shanghai Children’s Medical Center, School of Medicine, Shanghai Jiao Tong University, Shanghai, China

**Keywords:** SLE, Preterm infants, Premature comorbidities

## Abstract

**Background:**

Maternal systemic lupus erythematosus (SLE) is at greater risk of pregnancy complications and is associated with increased risk of preterm delivery. However hardly any study has looked at the influence of SLE on the outcomes of preterm infants. This study aimed to explore the influence of SLE on the outcomes of preterm infants.

**Methods:**

In this retrospective cohort study, preterm infants born to mothers with SLE from Shanghai Children’s Medical Center during 2012 to 2021 were enrolled. Infants were excluded if they were died during hospitalization or has major congenital anomalies and neonatal lupus. Exposure was defined as mother diagnosed SLE before or during pregnancy. Maternal SLE group was matched with Non-SLE group by gestational age, birth weight and gender. Clinical data has been extracted from patients’ records and registered. Major morbidities of premature and biochemical parameters in the two groups were compared using multiple logistic regression.

**Results:**

One hundred preterm infants born to 95 mothers with SLE were finally enrolled. The mean (standard deviation) of gestational age and birth weight were 33.09 (7.28) weeks and 1768.50 (423.56) g respectively. There was no significant difference in major morbidities between SLE group and non-SLE group. Compared with non-SLE group, SLE off-spring had significantly lower leukocytes, neutrophiles after birth, neutrophils and platlet in one week (mean difference: -2.825, -2.001, -0.842, -45.469, respectively). Among SLE group, lower birth weight and smaller gestational age were observed in SLE mothers with disease active during pregnancy, kidney involved, blood system involved and not taking Aspirin during pregnancy. In the multivariable logistic regression analysis, exposure to aspirin during pregnancy reduced the risk of very preterm birth and increased the incidence of survive without major morbidities among preterm infants born to SLE mothers.

**Conclusion:**

Born to mothers with SLE may not increase the risk of major premature morbidities, but the hematologic profile of SLE preterm infants may be different from preterm infants born to women without SLE. The outcome of SLE preterm infants is associated with maternal SLE status and may benefit from maternal aspirin administration.

**Supplementary Information:**

The online version contains supplementary material available at 10.1186/s13052-023-01436-5.

## Background

Systemic lupus erythematous (SLE) is an autoimmune disease that involves multiple organs and develops mainly in women of reproductive age. In the last decades, owing to improvements in multidisciplinary perinatal management, SLE was no longer viewed as a contraindication for pregnancy [1]. With the increase of pregnancy in women with SLE, numerous studies have reported that SLE is associated with increased risk of fetal complications including fetal loss, preterm delivery (20.8-28.7%)^2,3,4,5^, and intrauterine growth retardation.

Such studies have focused on the pregnancy outcomes of SLE [6–9] while there is only very few observational studies have assessed the outcome in preterm offspring of mothers with SLE [10]. Furthermore, pregnant women with SLE are at greater risk of thrombosis, infection, preeclampsia and multiple drugs would administrate during period of pregancy [11, 12], but hardly any study have looked at the influence of SLE on the outcome of preterm infants.

Understanding the outcomes of preterm infants born to SLE populations can inform clinical care and potentially provide mechanistic insight into such obstetric complication. Accordingly, the primary objective of this study is to assess the impact of SLE on premature newborns by comparing demographic data, prenatal and postnatal characteristics, laboratory data, and morbidities in a cohort of premature infants born to mothers with and without SLE. The secondary objective is to assess the influence of active SLE on preterm birth infants among infants with maternal SLE.

## Materials and methods

### Study design, setting and participants

This retrospective cohort study was carried out from February 2012 to May 2021 in the Neonatology Department of Shanghai Children’s Medical Center in Shanghai, China. Infants with preterm birth were included in this study. Infants with maternal SLE were identified from the database of medical records in our neonatology department born between February 2012 and May 2021. The exclusion criteria include: death during hospitalization, neonatal lupus and major congenital anomalies. The study participants were followed up until discharge.

### Data collection

Data of all preterm participants were collected by trained data abstractor in Shanghai Children’s Medical Center. All SLE mothers in this study delivered in Ren Ji Hospital which located next to our hospital. Ren Ji Hospital is the largest prenatal center of both autoimmune disease and high-risk pregnancy in Shanghai. Almost all pregnant women with SLE in this region will give birth in Renji Hospital. Maternal history were collected by trained data abstractor in Ren Ji Hospital. Site investigators were responsible for data quality control in both hospitals. Data were collected using the same definition in both hospitals.

### Exposure

SLE was diagnosed according to the revised criteria for the classification of SLE developed by the American College of Rheumatology [13] The non-SLE group was randomly selected from preterm infants admitted in our center. Non-SLE group were 1:2 matched with SLE group based on gender, birth weight (BW) ± 300 g, gestational age (GA) ± 6 days and date of birth ± 6 months. Infants in non-SLE group with major congenital anomalies, confirmed intrauterine infection, death during hospitalization and born to mother with other autoimmune diseases or malignant disease were excluded. The disease activity of SLE was made an appropriate evaluation based on the SLE Disease Activity Index 2000 (SLEDAI-2 K) [14].

### Outcome

The primary outcome was defined as infants who survived without major morbidities. Major morbidities of premature included intraventricular hemorrhage (IVH) (grade ≥ 3) and/or cystic periventricular leukomalacia (PVL), necrotizing enterocolitis (NEC) (stage ≥ 2), sepsis, Retinopathy of prematurity (ROP) (stage ≥ 3), and bronchopulmonary dysplasia (BPD). IVH was defined as greater than or equal to grade 3 according to the Papile criteria [15]. Cystic PVL was defined as the presence of periventricular cysts identified on cranial ultrasonography or magnetic resonance imaging. NEC was defined according to Bell criteria [16, 17]. Sepsis was defined as positive blood or cerebrospinal fluid culture (Exception for specimen contamination) [18]. Retinopathy of prematurity was diagnosed according to the International Classification of Retinopathy of Prematurity [19]. Bronchopulmonary dysplasia was defined as treatment with FiO_2_ > 0.21 for at least 28 days plus failure of room air challenge test at 36 weeks’ postmenstrual age [20]. Secondary outcome included complete blood count, total bilirubin (TBil) and alanine aminotransferase (ALT).

### Definitions of other covariates

Pregnancy-induced hypertension (PIH) was defined as an increase in blood pressure to ≥ 140/90 mmHg on at least two occasions ≥ 6 h apart that arises de novo after the 20th week of pregnancy. Preeclampsia (PE/E) was defined as pregnancy-induced hypertension with proteinuria > 0.3 g/L/d in the absence of a urinary tract infection or the abrupt onset of hypertension and proteinuria after 20 weeks of gestation. Seizures were required for a diagnosis of eclampsia. Gestational diabetes (GDM) was defined as any degree of glucose intolerance with onset or first recognition during pregnancy. HELLP (hemolysis, elevated liver enzymes and low platelet count) syndrome was defined as presence of hemolysis, high levels of lactate dehydrogenase or total bilirubin > 12 mg/L, elevated alanine aminotransferase levels of greater than twofold the upper normal value, and thrombocytopenia < 100*10^9^/L. Small for gestational age (SGA) was defined as infants whose weight was lower than the lower 10% limit of the CI of the Fenton curve. An Apgar score > 7 was defined as normal, while a score ≤ 7 was considered indicative of moderate or severe hypoxia [21].

### Statistical analysis

Normally distributed continuous data were expressed as mean and standard deviation (SD) and differences between two groups were tested by independent t-test. Categorical variables were presented with frequency and percentage and using chi-square test to exam the differences among groups.

In order to determine the association of neonatal outcome with maternal SLE in preterm birth, we performed multiple logistic regression analysis after adjustment of 1 min Apgar score and delivery type. Multiple linear regression was also performed in order to determine the association between maternal SLE and biochemical parameters. Log-transformation was conducted if biochemical parameters were not normally distributed before conducting linear regression.

Bi-variate analysis of baseline and neonatal outcome was conducted between active SLE and non-active SLE during pregnancy. In order to further investigate the effect of active SLE during pregnancy on neonatal outcome among infants born to maternal SLE, Multiple logistic regression analysis was conducted after adjustment of potential confounders. Each variable which has p-value < 0.1 was entered into the multivariable logistic regression model to control possible confounders. All tests were two-tailed, and p < 0.05 was considered statistically significant. Statistical analysis was performed using SPSS 22.0 and R 4.1.2.

### Ethics statement

This study protocol was reviewed and approved by the Ethics Board of Shanghai Children’s Medical Center, School of Medicine, Shanghai Jiao Tong University (SCMCIRB-K2021016-1). Written informed consent was obtained from all participants’ legal guardian. Approval to obtain clinical data from the database was received from the office of the medical director of the hospital. All patient information was kept confidential.

## Results

### Clinical characteristics of SLE mothers

One hundred and two preterm infants born to SLE mothers were found from the clinical database. One died during hospitalization, 1 diagnosed with neonatal lupus syndrome were excluded. The remaining 100 preterm infants were born to 95 mothers with SLE. The mean maternal age was 30.63 years with 6.61 years of disease duration. Disease active occurred in 46.3% of pregnancies. Renal manifestation was most common in these mothers (44.2%). During pregnancy, 91 mothers (95.8%) were taking steroids, 67 mothers (70.5%) were taking hydroxychloroquine (HCQ), 16 mothers (16.8%) were taking cytotoxic drugs including azathioprine and cyclosporine, and 75 mothers (78.9%) were taking aspirin. Mean positive antibodies were confirmed in mothers at onset of pregnancy as follows; anti-SSA/Ro antibody (47.9%), anti-SSB/La antibody (10.6%), dsDNA antibodies (57.4%) and antiphospholipid antibodies (aPLs) (5.3%). 57.9% mothers had pregnancy complications. Pregnancy induced hypertension was the most common complication in this cohort (37%). Characteristics of 95 SLE mothers are shown in Table 1.

**Table 1:** Clinical characteristics of 95 mothers with SLE

Variables	Value or no.(%)
Mean maternal age (yr, mean ± SD)	30.63 ± 4.09
Interval between diagnosis of SLE(yr, mean ± SD) SLE active during Pregnancy	6.61 ± 4.84 44(46.3%)
**Previous manifestations** Hematological disease Cutaneous lesions Articular disease Renal disease Serositis Neuropsychiatric symptoms	22(23.2%) 8(8.4%) 7(7.4%) 42(44.2%) 4(4.2%) 0(0%)
**Medication taking during pregnancy** Steroids HCQ Cytotoxic drugs Aspirin	91(95.8%) 67(70.5%) 16(16.8%) 75(78.9%)
**Positive antibodies at onset of pregnancy** ^**#**^ Anti-SSA/Ro Anti-SSB/La Anti-dsDNA Anti-Sm aPLs	45(47.9%) 10(10.6%) 55(57.4%) 7(7.4%) 5(5.3%)
**Pregnancy complications** PE/E GDM PIH HELLP	55(57.9%) 31(32.6%) 8(8.4%) 37(38.9%) 24(25.3%)
**Delivery mode** Vaginal delivery Cesarean delivery	8.4% (8) 91.6% (87)

^#^There is one missing data of Positive antibodies at onset of pregnancy

### Major morbidities and biochemical parameters: comparison between SLE group and non-SLE group

According to the matching strategy described previously, 98 infants born to mothers with SLE (SLE group) were matched to 196 preterm infants born to mothers without SLE (non-SLE group). The clinical characteristics of two groups were listed in Table 2.

**Table 2:** Comparison of major morbidities and biochemical parameters between SLE group and non-SLE group

Variables	SLE group	Non-SLE group	Total	P value
Number	98	196	294	
Gestational age(weeks)	33.13 ± 2.06	33.15 ± 2.17	33.14 ± 2.13	0.925
Birth weight(g)	1769.69 ± 423.94	1811.46 ± 408.90	1797.54 ± 413.72	0.415
SGA	36 (36.7%)	52 (26.5%)	88(29.9%)	0.072
Cesarean section	90 (91.8%)	154 (78.5%)	244(83.0%)	0.004**
1 min Apgar ≤ 7	11(11.2%)	39 (19.9%)	50(17.0%)	0.062
5 min Apgar ≤ 7	1(1.0%)	10(5.6%)	11(3.7%)	0.082
Morbidity				
BPD	15(15.3%)	41(20.9%)	56(19%)	0.250
IVH/PVL	0(0%)	2(1.0%)	2(0.7%)	0.997
ROP	1(1.0%)	0(0%)	1(0.3%)	0.995
NEC	2(2.0%)	7(3.6%)	9(3.1%)	0.478
Sepsis	0(0%)	5(2.6%)	5(1.7%)	0.997
Survival without major mobidites	82(83.7%)	147(75%)	229(77.9%)	0.093
Congenital heart disease^#^	50(71.4%)	96(73.8%)	146(73.0%)	0.713
Biochemical parameters				
Day one				
Leukocyte(*10^9/L)	9.47 ± 4.69	12.18 ± 7.20	11.28 ± 6.59	0.001**
Neutrophile(*10^9/L)	4.55 ± 2.61	6.44 ± 3.94	5.82 ± 3.66	0.000**
Platlet(*10^9/L)	226.70 ± 77.29	224.52 ± 76.31	225.25 ± 76.51	0.818
Hemoglobin(g/L)	173.20 ± 23.08	171.98 ± 28.92	172.39 ± 27.08	0.715
One week				
Leukocyte(*10^9/L)	10.08 ± 3.46	12.16 ± 13.45	11.47 ± 11.20	0.133
Neutrophile(*10^9/L)	3.97 ± 2.13	4.74 ± 3.19	4.48 ± 2.90	0.014*
Platlet(*10^9/L)	221.14 ± 99.24	265.15 ± 110.55	250.48 ± 108.75	0.001**
Hemoglobin(g/L)	161.75 ± 34.43	159.70 ± 39.57	160.38 ± 37.88	0.663
Lowest Hemoglobin(g/L)	126.16 ± 34.15	118.72 ± 33.54	121.20 ± 33.87	0.076
Lowest Platlet(*10^9/L)	172.42 ± 81.01	177.23 ± 79.08	175.63 ± 79.62	0.626
TBil max(U/L)	146.38 ± 47.35	139.70 ± 55.27	141.93 ± 52.77	0.307
ALT max(umol/L)	21.94 ± 37.79	20.66 ± 31.67	21.08 ± 33.77	0.759

^#^In our clinical practice, we only condut echocardiography examination on the neonates with positive CHD screening

*P＜0.05, **P＜0.01

The intact survival rate was 83.7% in the SLE group, and 75% in the non-SLE group, there was no statistic difference between the two groups. Besides no significant difference was found in the incidence of BPD, PVL, ROP, NEC, Sepsis nor in CHD (congenital heart disease) respectively between the two groups (Table 2).

Neonatal lupus erythematosus presents with 35% hematological abnormalities including anemia, neutropenia or thrombocytopenia and 15–25% asymptomatic elevation of aminotransferases, cholestasis, or hepatomegaly/splenomegaly due to the maternal antibodies exposure [22]. Thus we further compared clinical biochemical parameters between two groups. Compared with non-SLE group, Leukocyte and Neutrophile were significantly lower in SLE group at day 1. After one week, there was no difference in leukocyte between the two groups, but neutrophils in the SLE group was still significantly lower (p = 0.014), and platelet were also significantly lower (p = 0.001) than non-SLE group. No significant differences were observed in Hemoglobin, maximum ALT and TBil (Table 2).

Multivariate logistic regression of relationship between maternal SLE and outcomes of premature infants showed same result when delivery mode and 1 min Apgar score were included as covariates (Tables 3 and 4).

**Table 3:** Multivariate logistic regression of relationship between maternal SLE and morbidities of premature infants

Morbidity	OR	95%CI	*P*-value
BPD	0.736	0.375–1.445	0.373
Survival without major mobidites	0.590	0.310–1.122	0.108
Congenital heart disease	1.057	0.541–2.067	0.87

Maternal SLE, 1 min Apgar score and delivery mode were included in this model

**Table 4:** Multivariate linear regression of relationship between maternal SLE and Biochemical parameters of premature infants

Biochemical parameters	Mean Difference	95%CI	*P*-value
Day one			
Leukocyte(*10^9/L)	-2.825	-4.421–1.230	0.001**
Neutrophile(*10^9/L)	-2.001	-2.872–1.129	0.000**
Platlet(*10^9/L)	1.413	-17.327-20.152	0.883
Hemoglobin(g/L)	-0.019	-6.611-6.573	0.995
One week			
Leukocyte(*10^9/L)	-1.732	-4.471-1.007	0.215
Neutrophile(*10^9/L)	-0.842	-1.552–0.132	0.02**
Platlet(*10^9/L)	-45.469	-71.603–19.335	0.001**
Hemoglobin(g/L)	2.456	-6.845-11.757	0.605
Lowest Hemoglobin(g/L)	6.484	-1.650-14.619	0.118
Lowest Platlet(*10^9/L)	-6.401	-25.692-12.890	0.515
TBil max(U/L)	9.280	-3.632-22.191	0.159
ALT max(umol/L)	1.320	-6.849-9.489	0.751

Maternal SLE, 1 min Apgar score and delivery mode were included as covariates in this model

**P＜0.01

### Maternal influence on the outcomes of SLE preterm offspring

**Fig. 1:**
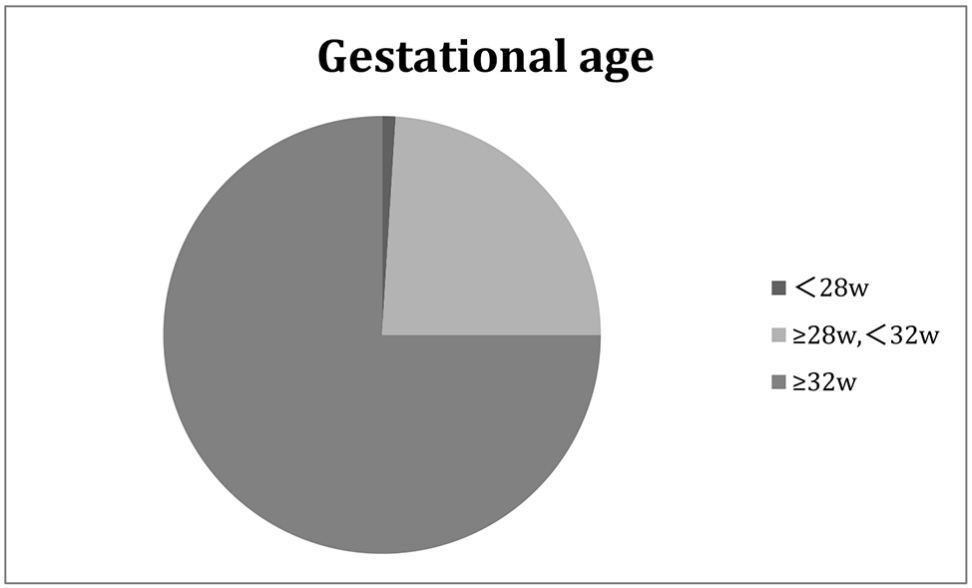
Gestational age distribution diagram of SLE preterm offspring

**Fig. 2:**
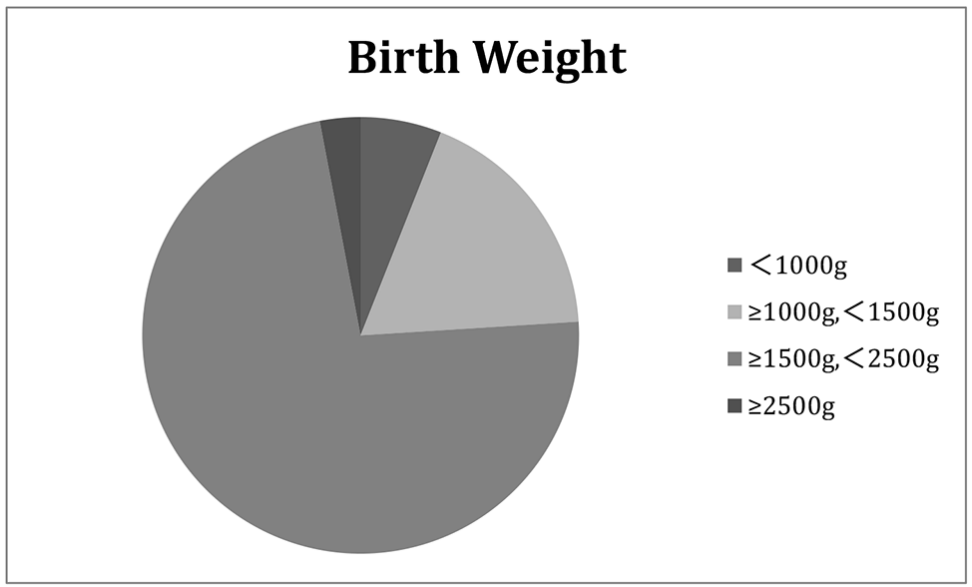
Birth weight distribution diagram of SLE preterm offspring

Among the 100 SLE preterm offspring, there were 52 males (52%) and 48 females (48%) including 5 pairs of twins. The mean GA of them was 33.09 ± 2.08 weeks, 1% was less than 28 weeks and 24% were between 28 and 32 weeks (Fig. 1). The mean birth weight was 1768.50 ± 423.56 g, 6% were less than 1000 g, 18% were between 1000 and 1500 g (Fig. 2). 36% were SGA.

We analyzed the influence of maternal status on SLE offspring which including SLE active, previous manifestations, medications exposure during pregnancy, positive antibodies and pregnancy complications. We found that SLE active during pregnancy, renal and blood system involvement, pregnancy complications, aspirin and HCQ administration were associated with offspring GA and birth weight. There was no difference in birth weight, gestational age and major morbidities between preterm infants of SLE mothers with positive aPLs and those with negative aPLs. (Supplementary Table 1). Univariate analysis displayed that very preterm birth (GA less than 32 weeks) was associated with blood systerm involvement, pregnancy complications and aspirin administration (Supplementary Table 2). In the multivariable logistic regression analysis, exposure to aspirin during pregnancy reduced the risk of very preterm birth (OR = 0.249, p = 0.033) and pregnancy complications were increased the risk of very preterm birth (OR = 3.018, p = 0.043) (Table 5). Univariate analysis displayed that survive without major morbidities was only associated with aspirin administration (p = 0.014, OR = 4.248, 95%CI: 1.343–13.431) (Supplementary Table 3). Multivariable logistic regression analysis also indicated that only aspirin administration during pregnancy were associated with survive without morbidities (p = 0.013) (Table 6).

**Table 5:** Multivariate logistic regression analysis of risk factors associated with very preterm birth among SLE group

Variables	OR	95%CI	*P-*value
SLE active during pregnancy	0.678	0.193–2.376	0.543
Blood system involvement	2.524	0.787–8.095	0.119
dsDNA^#^	1.603	0.547-4.700	0.390
Aspirin administration during pregnancy	0.249	0.070–0.892	0.033*
Complications	3.018	1.037–8.784	0.043*

^#^There is one missing data of Anti-dsDNA

*P＜0.05

**Table 6:** Multivariate logistic regression analysis of factors associated with preterm survive without major morbidities among SLE group

Variables	OR	95%CI	*P-*value
SLE active during pregnancy	4.688	0.863–25.473	0.074
SSB^#^	0.426	0.093–1.948	0.271
Aspirin administration during pregnancy	9.773	1.737–54.997	0.010*
Complications	0.315	0.092–1.074	0.065

^#^There is one missing data of Anti-SSB

*P＜0.05

**Fig. 3:**
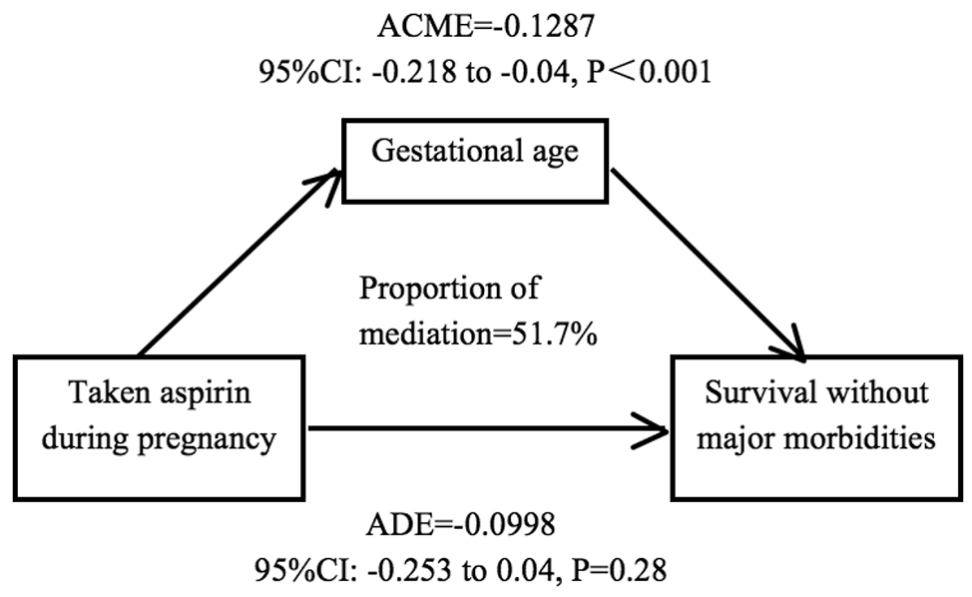
Schematic model of gestational age as the mediator between taking aspirin during pregnancy and survival without major morbidities in SLE group

We further tested whether the association between taking aspirin and survival without major morbidities was mediated by GA. Firstly univariate logistic regression result showed that there was no significant relationship between maternal taking aspirin and survival without major morbidities in very preterm infants. Then we conducted mediation analyses. As shown in Fig. 3, the results of mediation analyses showed that the total effect of maternal taking aspirin on survival without major morbidities was significant (B=-0.226, p < 0.001). Besides, the average causal mediation effects (ACME) between maternal taking aspirin and survival without major morbidities through gestatinal age was − 0.121 (p < 0.001), and the 95% bias-corrected bootstrap confidence interval was − 0.221 to -0.05, which indicated that the indirect effect of maternal taking aspirin on survival without major morbidities was statistically significant. In addition, the direct effect of maternal taking aspirin on survival without major morbidities (ADE=-0.105, p = 0.16) was not significant, indicating that GA mediated the relationship between maternal taking aspirin on survival without major morbidities. Univariate logistic regression analysis showed that taking aspirin was not associated with intact survival rate in the subgroup of very preterm infants (Supplementary Table 4).

## Discussion

In this retrospective cohort study, we found that there was no significant difference of major morbidities between SLE group and non-SLE group. However, SLE preterm offspring showed a hematologic profile that is different from that of non-SLE preterm offspring, with lower leukocyte counts, lower neutrophile and lower platlet.

The intact survival rate was 83.7% and the average GA was 33 weeks in SLE group, only 1% premature infants had a GA less than 28 weeks of which had most morbidities. No significant difference was found in each single morbidity including BPD, PVL, ROP, NEC, and Sepsis between the two groups. Mei-Ying et al. reported that SLE preterm offspring had much higher rate of neonatal infection as compared to preterm babies from women without SLE (47.62% vs. 16.81%)^11^. In their study the mean GA was 33 weeks plus 4 days which was similar as our study subject, the definition of sepsis in our study was positive blood or cerebrospinal fluid culture while their definition of neonatal infection was covering a wider range. Our definition was more precise, the broad definition may lead to bias. Furthermore, the non-SLE group they enrolled was all other premature infants delivered during the same time period, whereas our study matched the individuals’ GA and weight, excluding the confounding facts of varied GA and weight.

In our study hematological abnormalities were rare in both groups, however significant lower leukocyte, neutrophile and platelets were found in SLE preterm offspring than in non-SLE group. Maria Gariup et al. found that SLE Offspring (mean age 14.9 years) showed an immune profile that is different from relevant health control, with lower leukocyte counts and higher levels of mainly proinflammatory cytokines [23]. Besides their study showed SLE offspring had significantly higher proportions of history of nonallergic autoimmune conditions, and asthma was more frequent in SLE offspring than in health control. A cohort of 719 SLE offspring revealed that instead of rheumatic autoimmune disease, children born to women with SLE had an increased risk of non-rheumatic autoimmune diseases [24]. All these suggested that SLE offspring may have proinflammatory and autoimmune activation. The alteration in immune profile of SLE offspring seems to last for a long duration after birth while the maternal antibodies including Anti-SSA and anti-SSB in the offspring circulation are progressively reduced after birth and diappeared at about 12-month [25]. This phenomenon cannot be simply explained by maternal antibodies directed against auto-antigens in the fetal blood stream. Studies indicate that multiple molecular as well as cellular components originating in pregnant women are transferred to the fetus and program the fetal immune system [26]. SLE pregnancy might modify these signals and may consequently alter immunity in early life and childhood. On the other hand, the previous studies have proven familial risks between rheumatic autoimmune diseases (RAIDs), and SLE was associated with four of five RAIDs [27]. Further studies have highlighted a genetic component in the onset of SLE and other autoimmune diseases that may lead to several different phenotypes even though the genotype is the same [28]. Many of the shared loci were related to immune processes, such as interferon signalling and polymorphisms of STAT genes that may have a wide spectrum of phenotype [28, 29].

The incidence of CHD in this study was much higher than previous reports and with no difference from the non-SLE group. In a large population-based study conducted by Evelyne Yinet et al., reported that in comparison with children from the general population, children born to women with SLE have an increased risk of CHD 5.1% [95% CI, 3.7–7.1] versus 1.9% [95% CI, 1.6–2.2] [30]. One potential reason for these differences in the incidence of CHD is that the GA was significant different between two studies. The mean GA of SLE offspring in their study was 37.7 weeks which was significant different from control children whose mean GA was 38.8 weeks. Epidemiologic study suggests that premature neonates have a more than 2-fold higher risk of cardiovascular abnormalities [31]. CHD is more likely to be associated with prematurity instead of SLE.

In our study the maternal age, SLE disease duration, SLE active, and antibodies characteristics were similar as several studies previously reported in Asia [4, 32–34]. The incidence of pregnancy complications in this cohort was significantly higher than other SLE cohorts. The results of our study showed that PIH and preeclampsia are the significant problems in SLE pregnancies with preterm infants. In our study, nearly one-third of SLE mothers were complicated with PIH and preeclampsia, whereas according to previous studies only 3.1–19.2% of the pregnancies in SLE women were affected by such conditions [4, 35]. Several studies have explored that PIH can significantly increase the risk of preterm birth [36], while our subjects are all preterm infants. Yen-Ju Chen et al. has reported that preterm risks increased markedly in participants with both preeclampsia/eclampsia and SLE (OR: 17.5, 95% CI: 12.6_24.1, p < 0.01)^32^. Therefore, the high incidence of PIH and preeclampsia in our cohort is understandable and we speculate that SLE pregnant women complicated with PIH/PE are more likely to give birth prematurely than those SLE mothers without PIH/PE.

The rate of cesarean section in SLE group was significantly higher than non-SLE group in this study (91.8% vs. 78.5%). Jae-kyoon Hwang et al. reported a slightly lower cesarean section rate by 84.8% in preterm SLE offspring [37]. In SLE women, the general cesarean rate was previously reported to be 30-40%^2,38,39,40^. In our study, high cesarean section rate could be the confounding factor of SLE, premature birth and high rate of PIH/PE.

Further analysis in this study showed that maternal status including SLE active during pregnancy, renal and blood system involvement and pregnancy complications were associated with smaller offspring GA and lower offspring birth weight. Studies have showed that maternal SLE activity in the last 6–12 months or at conception increased the risk of maternal disease activity (subsequent flare during pregnancy and puerperium) and hypertensive complications, fetal morbidity and mortality [41, 42]. Considering the high risk of active SLE during pregnancy, EULAR (European League Against Rheumatism) recommends that SLE patients plan their pregnancies at least 6 months after achieving remission [42].

Taking aspirin during pregnancy was associated with larger offspring GA and higher offspring birth weight in this study, besides analysis also indicated that aspirin administration during pregnancy would increase the rate of survival without major morbidities. Because of the significant risk of preeclampsia in all lupus pregnancies, especially those with preexisting renal impairment or active lupus, low-dose aspirin is indicated in all women with SLE starting around 12 weeks gestation [43]. There is no study reported the long-term effect of aspirin on offspring outcomes. Here according to the mediation model, GA interaction can explain the effect of aspirin on survival without major morbidities 51.7% which means aspirin reduces morbidities of preterm infants by increasing gestational weeks. Besides, univariate logistic regression analysis showed that in very preterm infants of taking aspirin during pregnancy with survive without major morbidities in among SLE group.

To our knowledge, this was the first retrospective cohort study based on nearly 10 years clinical data of preterm infants born to mothers with SLE. Additionally almost every key influence of the SLE pregnancy on preterm infants was included in this study. Nevertheless, there were several limitations to our study. Firstly, the sample size of this retrospective study is not very large and the actual sample size for preterm morbidities comparison was considerably small, however, the sample size of this study is one of the largest sample sizes of all studies at present. Secondly, in order to control confounding factors, the matching criteria in this study are relatively strict, we can only achieve 1: 2 matching. Finally, the end point of our study was discharge. The long-term outcome is very important for preterm infants. In the future, we may carry out a prospective cohort study of preterm offspring of SLE.

## Conclusion

Born to mothers with SLE may not increase the risk of major premature comorbidities, but the hematologic profile of SLE preterm infants may be lower than preterm infants born to women without SLE. The outcome of preterm infants is associated with maternal SLE status and may benefit from maternal aspirin administration.

## Electronic supplementary material

Below is the link to the electronic supplementary material.

Supplementary Material 1

Supplementary Material 2

Supplementary Material 3

Supplementary Material 4

Supplementary Material 5

Supplementary Material 6

## Data Availability

The original contributions presented in the study are included in the article/supplementary material, futher inquiries can be directed to the corresponding authors.
