# Supplementary material for: Association of preterm outcome with maternal systemic lupus erythematosus: a retrospective cohort study
Source: Ital J Pediatr. 2023 Apr 1;49:43. doi: 10.1186/s13052-023-01436-5 (PMC10068147; doi:10.1186/s13052-023-01436-5)
Supplement: Supplementary file 1 — Supplementary Material 1 [file 13052_2023_1436_MOESM1_ESM.docx]

**Supplementary Table 1 Maternal influence on gestational age and birth weight among SLE group**

|  | case | Gestational age (weeks) | Birth weight (g) | *t*  GA  BW | *p*  GA  BW |
| --- | --- | --- | --- | --- | --- |
| SLE active during pregnancy |  |  |  |  |  |
| No | 55 | 33.51±1.86 | 1882.09±393.57 | 2.282 | 0.025* |
| Yes | 45 | 32.58±2.24 | 1629.67±421.55 | 3.090 | 0.003* |
| Renal involvement |  |  |  |  |  |
| No | 58 | 33.47±1.80 | 1851.38±383.36 | 2.081 | 0.041* |
| Yes | 42 | 32.57±2.33 | 1654.05±453.58 | 2.315 | 0.021* |
| Blood systerm involvement |  |  |  |  |  |
| No | 77 | 33.33±1.94 | 1823.44±417.44 | 2.12 | 0.036* |
| Yes | 23 | 32.30±2.37 | 1584.57±399.24 | 2.432 | 0.017* |
| Compications |  |  |  |  |  |
| Without | 55 | 33.57±1.72 | 1895.09±382.79 | 2.535 | 0.013* |
| With | 45 | 32.51±2.34 | 1613.78±423.44 | 3.485 | 0.001* |
| Aspirin administration |  |  |  |  |  |
| No | 20 | 32.14±2.26 | 1575.00±461.11 | -2.350 | 0.021* |
| Yes | 80 | 33.33±1.97 | 1816.88±402.28 | -2.335 | 0.022* |
| HCQ administration |  |  |  |  |  |
| No | 29 | 32.65±1.89 | 1629.66±413.43 | -1.362 | 0.176 |
| Yes | 71 | 33.27±2.14 | 1825.21±417.26 | -2.132 | 0.035* |
| aPLs |  |  |  |  |  |
| Negative | 94 | 33.06±2.11 | 1773.83±429.25 | -0.257 | 0.798 |
| Positive | 5 | 33.31±1.84 | 1677.00±380.19 | 0.494 | 0.623 |
|  |  |  |  |  |  |

*P＜0.05
