# Supplementary material for: Association of preterm outcome with maternal systemic lupus erythematosus: a retrospective cohort study
Source: Ital J Pediatr. 2023 Apr 1;49:43. doi: 10.1186/s13052-023-01436-5 (PMC10068147; doi:10.1186/s13052-023-01436-5)
Supplement: Supplementary file 2 — Supplementary Material 2 [file 13052_2023_1436_MOESM2_ESM.docx]

**Supplementary Table 2.** **Univariate logistic regression analysis of risk factors associated with very preterm birth among SLE group**

| Variables | OR | 95%CI | *P-*value |
| --- | --- | --- | --- |
| SLE active during Pregnancy | 2.250 | 0.893-5.668 | 0.085 |
| Previous manifestations |  |  |  |
| Hematological disease | 3.179 | 1.171-8.633 | 0.023* |
| Cutaneous lesions | 1.909 | 0.422-8.637 | 0.401 |
| Articular disease | 0.845 | 0.164-4.359 | 0.840 |
| Renal disease | 2.136 | 0.853-5.350 | 0.105 |
| Serositis | 1.000 | 0.099-10.073 | 1.000 |
| Medication exposures during pregnancy |  |  |  |
| Steroids | 1.000 | 0.099-10.073 | 1.000 |
| HCQ | 1.067 | 0.391-2.915 | 0.899 |
| Cytotoxic drugs | 0.908 | 0.267-3.093 | 0.878 |
| Aspirin | 0.231 | 0.081-0.653 | 0.006* |
| Positive antibodies at onset of pregnancy |  |  |  |
| Anti-SSA/Ro | 0.505 | 0.198-1.287 | 0.152 |
| Anti-SSB/La | 1.125 | 0.274-4.617 | 0.870 |
| Anti-dsDNA | 2.436 | 0.910-6.523 | 0.076 |
| aPLs | 0.729 | 0.078-6.848 | 0.782 |
| Pregnancy complications | 2.820 | 1.102-7.216 | 0.031* |
| PE/E | 2.721 | 1.061-6.975 | 0.037* |
| GDM | 1.000 | 0.189-5.304 | 1.000 |
| PIH | 2.302 | 0.915-5.791 | 0.076 |
| HELLP | 0.583 | 0.065-5.246 | 0.631 |

*P＜0.05
