# Supplementary material for: Association of preterm outcome with maternal systemic lupus erythematosus: a retrospective cohort study
Source: Ital J Pediatr. 2023 Apr 1;49:43. doi: 10.1186/s13052-023-01436-5 (PMC10068147; doi:10.1186/s13052-023-01436-5)
Supplement: Supplementary file 3 — Supplementary Material 3 [file 13052_2023_1436_MOESM3_ESM.docx]

**Supplementary Table 3.** **Univariate logistic regression analysis of factors associated with preterm survive without major morbidities among SLE group**

| Variables | OR | 95%CI | *P-*value |
| --- | --- | --- | --- |
| SLE active during Pregnancy | 1.062 | 0.362-3.119 | 0.913 |
| Previous manifestations |  |  |  |
| Hematological disease | 0.877 | 0.253-3.035 | 0.836 |
| Cutaneous lesions | 1.364 | 0.156-11.908 | 0.779 |
| Articular disease | 0.636 | 0.120-3.385 | 0.596 |
| Renal disease | 0.918 | 0.312-2.701 | 0.877 |
| Serositis | 0.556 | 0.054-5.706 | 0.621 |
| Medication exposures during pregnancy |  |  |  |
| Steroids | 1.800 | 0.175-18.487 | 0.621 |
| HCQ | 1.136 | 0.357-3.619 | 0.829 |
| Cytotoxic drugs | 0.867 | 0.218-3.446 | 0.839 |
| Aspirin | 4.248 | 1.343-13.431 | 0.014* |
| Positive antibodies at onset of pregnancy |  |  |  |
| Anti-SSA/Ro | 0.930 | 0.319-2.713 | 0.895 |
| Anti-SSB/La | 0.276 | 0.070-1.089 | 0.066 |
| Anti-dsDNA | 0.538 | 0.172-1.686 | 0.288 |
| Anti-Sm | 1.169 | 0.131-10.424 | 0.889 |
| aPLs | 0.759 | 0.079-7.277 | 0.811 |
| Pregnancy complications | 0.429 | 0.142-1.289 | 0.132 |
| PE/E | 0.514 | 0.172-1.538 | 0.234 |
| PIH | 0.527 | 0.179-1.550 | 0.245 |
| HELLP | 0.949 | 0.103-8.714 | 0.963 |

*P＜0.05
