# Supplementary material for: Association of preterm outcome with maternal systemic lupus erythematosus: a retrospective cohort study
Source: Ital J Pediatr. 2023 Apr 1;49:43. doi: 10.1186/s13052-023-01436-5 (PMC10068147; doi:10.1186/s13052-023-01436-5)
Supplement: Supplementary file 4 — Supplementary Material 4 [file 13052_2023_1436_MOESM4_ESM.docx]

**Supplementary Table 4.** **Univariate logistic regression analysis of taking aspirin during pregnancy with survive without major morbidities in very preterm infants among SLE group**

| Variables | OR | 95%CI | *P-*value |
| --- | --- | --- | --- |
| Taking aspirin during Pregnancy | 2.250 | 0.439-11.522 | 0.330 |
