# Supplementary material for: Association of preterm outcome with maternal systemic lupus erythematosus: a retrospective cohort study
Source: Ital J Pediatr. 2023 Apr 1;49:43. doi: 10.1186/s13052-023-01436-5 (PMC10068147; doi:10.1186/s13052-023-01436-5)
Supplement: Supplementary file 5 — Supplementary Material 5 [file 13052_2023_1436_MOESM5_ESM.docx]

26th, July 2022

Dear Editor,

We would like to submit the enclosed manuscript entitled "Association of preterm outcome with maternal systemic lupus erythematosus: a retrospective cohort study", which we wish to be considered for publication as an Original Research in Italian Journal of Pediatrics.

SLE pregnancy is at greater risk of pregnancy complications and is associated with increased risk of preterm delivery. However hardly any study has looked at the influence of SLE on the outcomes of preterm infants.

In the current study, we retrospectively analyzed the short outcomes of preterm SLE offspring over the past 10 years. The result showed that born to mothers with SLE may not increase the risk of major premature comorbidities, but the hematologic profile of preterm SLE offspring may be different from preterm infants born to women without SLE.

To our knowledge, this was the first retrospective cohort study of preterm infants born to mothers with SLE. Understanding the outcomes of preterm infants born to SLE populations can inform clinical care and potentially provide mechanistic insight into such obstetric complication.

We believe that this article is appropriate for publication by Italian Journal of Pediatrics, it would be of great interest to a broad range of readers including neonatologists, obstetrician, rheumatologists and other researchers in related fields.

Finally, we would like to make the following declarations: All authors of this paper have read and approved the final version submitted and contents of this manuscript have not been copyrighted or published previously and is not under consideration for publication elsewhere.

We deeply appreciate your consideration of our manuscript, and we look forward to receiving comments from the reviewers. If you have any queries, please don’t hesitate to contact me at the address below.
Thank you and best regards.

Yours sincerely,
Pro. Fei Bei, MD, PhD

Department of Neonatology, Shanghai Children's Medical Center, School of Medicine, Shanghai Jiao Tong University, Shanghai, China

Shanghai 200127,China

E-mail: beifei@scmc.com.cn
