# Supplementary material for: Association of preterm outcome with maternal systemic lupus erythematosus: a retrospective cohort study
Source: Ital J Pediatr. 2023 Apr 1;49:43. doi: 10.1186/s13052-023-01436-5 (PMC10068147; doi:10.1186/s13052-023-01436-5)
Supplement: Supplementary file 6 — Supplementary Material 6 [file 13052_2023_1436_MOESM6_ESM.docx]

Dear Editor,

**Resubmission of “Association of preterm outcome with maternal systemic lupus erythematosus: a retrospective cohort study' (ITJP-D-22-00388)**

We thank the editor and reviewers for the insightful comments on our manuscript. Below, we have provided point-by-point response to each comment. We have also revised our manuscript accordingly.

**Reviewer 1**
The Authors present the outcome of preterm infants, comparing SLE vs non-SLE mothers. They found a positive association between aspirin intake and survival without major comorbidities, and underline that  the SLE group was characterized by leuko-neutropenia at birth.
The work is interesting, only some concerns need to be addressed: The fact that aspirin intake is associated with the risk of very preterm birth and at the same time is protective against major morbidities is peculiar. As reported also by the Authors, the protective role of aspirin against pre-eclampsia is well established in SLE patients with previous renal involvement and/or antiphospholipid antibodies it is still debated whether aspirin is protective in patients without renal involvement or antiphospholipid antibodies (Tani C, Lupus Sci Med. 2022 Jun;9(1):e000714. )
The fact that in this work aspirin intake was associated with the risk of very preterm birth might depend on the disease activity of the disease of the mother, and to antiphoospholipid syndrome as well as to other factors. Please comment on that.
Please clarify if, in 78.9% of mothers taking aspirin, there was  any differenc between the mothers taking and those not taking aspirin.

**Response**: Thank you very much for your comments and advice. In this retrospective cohort study, exposure to aspirin during pregnancy was associated with larger gestational age among SLE mothers. In multivariable logistic regression analysis, mothers who took aspirin during pregnancy reduced the rate of very preterm birth (OR=0.249), and improve the rate of survive without major morbidities. We apologized for our inaccurate expression in the manuscript. We have revised the according sentence to address your concerns and hope that it is now clearer. We’ve changed this sentence as to [In the multivariable logistic regression analysis, exposure to aspirin during pregnancy reduced the risk of very preterm birth and increased the incidence of survive without major morbidities among preterm infants born to SLE mothers.] (page 2, line 53-56; page 7 line 234-241).

We further analyzed the differences in disease activity, antiphospholipid antibody and renal involvement between mothers who took and did not take aspirin. Below tables showed that aspirin intake was associated with less renal involvement, less SLE active. In multivariable logistic regression analysis, neither SLE active nor renal involvement were related to the very preterm birth. So aspirin intake was an independent protective factor of very preterm birth, which was not associated with mother SLE status.

|  |  | Renal involvement | | Totall | X² | *P-*value |
| --- | --- | --- | --- | --- | --- | --- |
|  |  | No | Yes |  |  |  |
| Aspirin administration during pregnancy | No | 5(25%) | 15(75%) | 20(21.1%) | 9.737 | 0.002 |
|  | Yes | 48(64%) | 27(36%) | 75(78.9%) |  |  |
|  | Totall | 53 | 42 | 95 |  |  |

|  |  | SLE active during Pregnancy | | Totall | X² | *P-*value |
| --- | --- | --- | --- | --- | --- | --- |
|  |  | No | Yes |  |  |  |
| Aspirin administration during pregnancy | No | 2(10%) | 18(90%) | 20(21.1%) | 19.443 | 0.000 |
|  | Yes | 49(65.3%) | 26(34.7%) | 75(78.9%) |  |  |
|  | Totall | 51 | 44 | 95 |  |  |

|  |  | aPL | | Totall | X² | *P-*value |
| --- | --- | --- | --- | --- | --- | --- |
|  |  | Negtive | Positive |  |  |  |
| Aspirin administration during pregnancy | No | 19(95%) | 1(5%) | 20(21.3%) | 0.005 | 0.943 |
|  | Yes | 70(94.6%) | 4(5.4%) | 74(78.7%) |  |  |
|  | Totall | 89 | 5 | 94 |  |  |

**Reviewer 2**
This is avery well written paper about a study on outcomes of preterm born to SLE mothers.This study analyzed the main differences between preterm born to SLE mother and non-SLE mother, excluding those who died during hospitalization, with major congenital abnormalities and with neonatal lupus. They did not evaluatesignificant differences among the two groups, however in the SLE group they evaluated significant differences between preterm babies born to mothers with active diseaserather than slow disease activity. Moreover they identified that patients may benefit from maternal administration.The abstract is enough concise and clearly identifies the main characteristics of the study and the results. They give a clear message useful for the readers.
Background is clear and gives enough information about the topic of the study.
The authors clearly identified the purposes of the study, designing a scale of importanceof the different purposes.
**Question 1:** The methodology of the study is appropriate and clearly described; however, it is not clear why the authors decided to exclude newborn died duringhospitalization and newborn with neonatal lupus. This last is one of the main complications of pregnancy in SLE. May you provide a clear explanation of this?

**Response**: Thank you very much for your time involved in reviewing the manuscript and your very encouraging comments on the merits. Neonatal lupus is a passively-acquired autoimmune disease, so far there were many case reports and cohort studies on neonatal lupus. While in this study, our study population was premature infants exposed to maternal lupus instead of neonatal lupus, whose outcomes are rarely reported. So we exclude the newborn with neonatal lupus and use cohort study by matching gender, birth weight and gestational age to see the influence of maternal SLE on the outcomes of preterm infants without neonatal lupus. The deceased case was also excluded because the death was due to the gave up of treatment.

**Question 2:** Although you decided to perform a retrospective study on the outcomes of preterm newborn born to SLE mothers, it would be interesting to know the proportion of offspring that are preterm out of the total number of newborns born to mothers with lupus.

**Response**: Thank you for this insightful comment. In a large Chinese SLE pregnant cohort study reported by Renji Hospital where all SLE mothers in our study gave birth, the proportion of preterm offspring among the total number of newborns born to mothers with lupus was 28.7%^4^, which was similar to that of other countries (20.8%-28.7%)^2,3,5^. We have added the proportion in the manuscript (page 3, line 74).

**Question 3:** Are there SLE mothers with positive antiphospholipid antibodies antiBeta2glicoprotein and anticardiolipin? If yes, please specify how many mothers were positive and if they influenced the outcomes of interest.

**Response**: Thank you for your insightful comments. There are five SLE mothers with positive antiphospholipid antibodies (Table 1). There was no difference in birth weight, gestational age and major morbidities between preterm infants of SLE mothers with positive antiphospholipid antibodies and those with negative antiphospholipid antibodies. We have added this result in the manuscript accordingly (page 7, line232-234; supplemental table 1-3).

**Question 4:** Have you evaluated differences in the brain ultrasounds or in the offspring birth weight?

**Response**: Thank you for point this out. We have evaluated differences in the brain ultrasounds. The major morbidities of intraventricular hemorrhage and/or cystic periventricular leukomalacia were diagnosed according to the results of brain ultrasound and MRI. We've also evaluated the differences in the offspring birth weight among SLE group. Result showed that SLE active during pregnancy, renal and blood system involvement, pregnancy complications were associated with lower birth weight, while aspirin intake during pregnancy was associated with higher offspring birth weight (Supplementary table 1).

**Question 5:** In discussion you stated that SLE offspring had an increased risk of non-rheumatic autoimmune diseases and SLE pregnancy might modify these signals and may consequently alter immunity in early life and childhood. However, in literature there are numerous studies that in the last few years have highlighted a genetic component in the onset of SLE and other autoimmune diseases that may lead to several different phenotypes even though the genotype is the same. An important example is the STAT family or interferonopathies that may have a wide spectrumof phenotype.
**Response:** We thank the reviewer for the instructive comments and agreed with the reviewer. We have revised the discussion section by focusing this important points as to [On the other hand, the previous studies have proven familial risks between rheumatic autoimmune diseases (RAIDs), and SLE was associated with four of five RAIDs^27^. Further studies have highlighted a genetic component in the onset of SLE and other autoimmune diseases that may lead to several different phenotypes even though the genotype is the same^28^. Many of the shared loci were related to immune processes, such as interferon signalling and polymorphisms of STAT genes that may have a wide spectrum of phenotype^28,29^.] (page 10, line 309-315).
